# The prediction of acute toxicity (LD_50_) for organophosphorus-based chemical warfare agents (V-series) using toxicology in silico methods

**DOI:** 10.1007/s00204-023-03632-y

**Published:** 2023-12-05

**Authors:** Maciej Noga, Agata Michalska, Kamil Jurowski

**Affiliations:** 1 Department of Regulatory and Forensic Toxicology, Institute of Medical Expertises in Łódź, ul. Aleksandrowska 67/93, 91-205 Łódź, Poland; 2 Institute of Medical Expertises in Łódź, ul. Aleksandrowska 67/93, 91-205 Łódź, Poland; 3 Laboratory of Innovative Toxicological Research and Analyzes, Institute of Medical Studies, Medical College, Rzeszów University, Al. mjr. W. Kopisto 2a, 35-959 Rzeszów, Poland

**Keywords:** Chemical warfare agents, Organophosphate, Nerve agents, Acute toxicity, Toxicology in silico

## Abstract

Nerve agents are organophosphate chemical warfare agents that exert their toxic effects by irreversibly inhibiting acetylcholinesterase, affecting the breakdown of the neurotransmitter acetylcholine in the synaptic cleft. Due to the risk of exposure to dangerous nerve agents and for animal welfare reasons, in silico methods have been used to assess acute toxicity safely. The next-generation risk assessment (NGRA) is a new approach for predicting toxicological parameters that can meet modern requirements for toxicological research. The present study explains the acute toxicity of the examined V-series nerve agents (*n* = 9) using QSAR models. Toxicity Estimation Software Tool (ver. 4.2.1 and ver. 5.1.2), QSAR Toolbox (ver. 4.6), and ProTox-II browser application were used to predict the median lethal dose. The Simplified Molecular Input Line Entry Specification (SMILES) was the input data source. The results indicate that the most deadly V-agents were VX and VM, followed by structural VX analogues: RVX and CVX. The least toxic turned out to be V-sub x and Substance 100A. In silico methods for predicting various parameters are crucial for filling data gaps ahead of experimental research and preparing for the upcoming use of nerve agents.

**Supplementary Information:**

The online version contains supplementary material available at 10.1007/s00204-023-03632-y.

## Introduction

Organophosphorus compounds have a relatively simple chemical structure, which should ease the taming of this group of compounds from both a chemical and toxicological point of view. However, organophosphates (OPs) remain a challenge for toxicologists due to their unique chemical structure, which implies a distinctive mechanism of action. One of the first problems with these compounds during toxicological risk assessment was related to the concept of the threshold of toxicological concern (TTC) concept, which was a risk assessment tool for substances present at low oral exposure and lacking hazard data previously developed by Cramer et al. (1976) and further extended by Munro et al. (2008) and Kroes and Kozianowski (2002) and Kroes et al. (2004). Previously, a threshold of 0.3 μg/kg bw/day was established for OPs based on the analysis of data from OP insecticides included in the Munro dataset. These insecticides belonged to the Cramer class III group with a threshold of 1.5 μg/kg bw/day. However, when the OPs were excluded from the Cramer class III group, the threshold for the remaining substances in this group was not recalculated. Through the ongoing reevaluation of the Cramer class III substances in the Munro dataset, new thresholds have been established for various compounds, including OPs with carbamates, organohalogens, and the remaining Cramer class III substances. These thresholds can be used effectively within the TTC concept. It is confirmed that the TTC threshold of 0.30 μg/kg bw/day for OPs remains valid, even when incorporating carbamates. Why do OPs cause so many problems? All this is due to the chemical structure determining the toxic properties.

From a chemical point of view, OPs are a class of organic chemicals derived from phosphoric acids and their derivatives, characterised by the presence of at least one carbon–phosphorus bond (Crofts 1958). Among the various types of phosphorus-containing compounds, pentavalent OPs (sp^3^d-type hybridisation of the valence orbitals of the phosphorus atom) find widespread usage in industrial (especially pesticides) (Worek et al. 2020) and war (chemical warfare agents) (Diauudin et al. 2019). The toxicity of these compounds is significantly influenced by the substituents attached to the phosphorus atom in the esters of phosphoric acids. One of the most intriguing and problematic chemical classes is organophosphorus chemical warfare agents (OP-CWAs), a distinct class of synthetic compounds recognised for their exceptionally high toxicity, surpassing that of various other chemical substances (Kloske and Witkiewicz 2019). One of the unique groups of OP-CWAs is the V-series compounds, which are considered very dangerous from a toxicological point of view. Still, there is limited experimental data about them. The chemical structures with CAS and SMILES notation of the known OP-CWAs V-agents are presented in Table 1.

**Table 1:** The chemical structures with CAS and SMILES notation of the known V-agents (belonging to organophosphorus chemical warfare agents)

Number	Acronym	Known structure	CAS	SMILES
(1)	VE (EA-1517)		21738–25-0	O = P(OCC)(SCCN(CC)CC)CC
(2)	VG (EA-1508)		78–53-5	O = P(OCC)(OCC)SCCN(CC)CC
(3)	VM (EA-1664)		21770–86-5	O = P(OCC)(SCCN(CC)CC)C
(4)	VR (RVX, Substance 33)		159939–87-4	O = P(OCC(C)C)(SCCN(CC)CC)C
(5)	VS (EA-1677)		73835–17-3	CCOP(= O)(CC)SCCN(C(C)C)C(C)C
(6)	V-sub x (GD-7, EA-5478)		556–75-2	O = P(OCC)(SCCSCC)C
(7)	VX (EA-1701)		50782–69-9	O = P(OCC)(SCCN(C(C)C)C(C)C)C
(8)	CVX (EA-6043)		468712–10-9	CCCCOP(= O)(C)SCCN(CC)CC
(9)	Substance 100A (EA-3148)		93240–66-5	O = P(OC1CCCC1)(SCCN(CC)CC)C

**Fig. 1:**
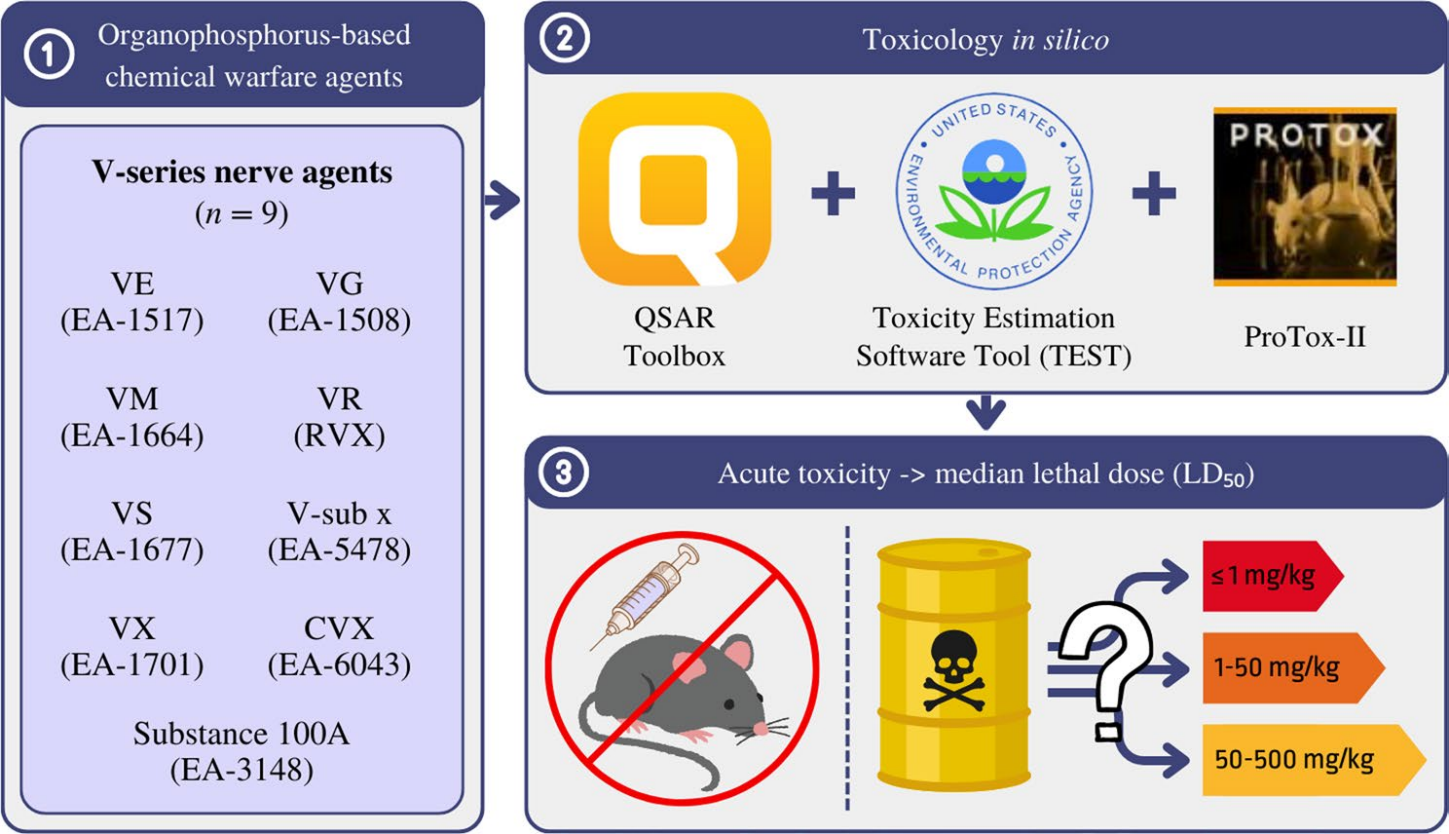
The schematic idea of predicting V-agent acute toxicity (LD_50_ rat, oral) using toxicology in silico methods

The median lethal dose (lethal dose 50; LD_50_) has been controversial among biologists and animal ethicists ever since it was introduced by Trevan in 1927 (Pillai et al. 2021). Toxicologists perennially employ the LD_50_ test as an initial step in evaluating the toxicity of a substance. However, animal ethicists raise concerns about LD_50_ tests due to the pain animals experience during these experiments, and they argue that the LD_50_ values obtained are unimportant. The LD_50_ test focuses explicitly on acute toxicity and determines the dose at which a substance becomes lethal to a percentage of the tested animals. Moreover, the LD_50_ test is not in line with the principles of replacement, reduction, and refinement of animal use and welfare (3R), which are principles aimed at minimising the use of animals in toxicity tests when applicable (Faria et al. 2016). In 2002, the Organisation for Economic Cooperation and Development (OECD) deleted the LD_50_ test as a requirement for testing new chemicals. The OECD replaced the classical LD_50_ test with three alternative tests: the fixed dose procedure (FDP), the acute toxic class method (ATC), and the up and down procedure (UDP). Although LD_50_ is no longer used as a relevant dose descriptor in modern toxicological health risk assessment, it is still an essential parameter, the absence of which for extreme poisons is a significant gap in the literature. The subject of OP-CWAs relationships is ancient, and we should know almost everything about them, but it is still a never-ending story (Bolt 2023). To fulfil the modern requirements for toxicological research in the twenty-first century and to consider the next generation risk assessment (NGRA) with a new approach to toxicity testing (i.e. taking into account the prediction of toxicological parameters first), it is necessary to apply in silico toxicology methods to eliminate unnecessary animal studies (Bolt and Hengstler 2020). Researching this parameter is essential to determine the accurate level of risk that V-agents may pose. The study aimed to predict the acute toxicity (LD_50_) for V-series NAs using modern toxicology in silico methods. The study was conducted using various in silico models included in the software: QSAR Toolbox (Dimitrov et al. 2016), Toxicity Estimation Software Tool (TEST) and ProTox-II. A general flow chart showing the acute toxicity parameter (LD_50_, rat, oral) estimation process is presented in Fig. 1.

## Methods

### Application of QSAR Toolbox

We conducted in silico analyses using the QSAR Toolbox (ver. 4.6) (Dimitrov et al. 2016; Schultz et al. 2018), a standalone software application recommended by the OECD. The QSAR Toolbox was developed by OASIS (Yordanova et al. 2019) in collaboration with the OECD and the European Chemicals Agency (ECHA) to evaluate the potential hazards of chemicals with in silico models. This scientific software uses a flexible workflow to fill data gaps by building compound categories and estimating incomplete data through read-across or local QSARs. In addition to read-across and trend analysis, the QSAR Toolbox includes numerous databases of experimental results. Acute toxicity was estimated using QSAR Toolbox software by manual categorisation and data gap filling method (Mombelli and Pandard 2021; Kutsarova et al. 2021a, b). The input data for the software were the SMILES of individual V-series NAs, included in Table 1. The target endpoint was defined as human health hazards, acute toxicity, LD_50_ (endpoint), oral (route of administration), and rats (test organisms/species). The categorisation was determined as ‘organic functional groups’. The read data were selected only for the initially targeted endpoint. The read-across method for ‘qualitative’ endpoints was used to fill data gaps. The scale/unit to estimate LD_50_ was chosen in mg/kg bw. Subcategorisation was then used to exclude structurally different prediction compounds from the investigated V-agents. Individual subcategories were made for each chemical. The initial stage of subcategorisation for the targeted nerve agents (NAs) had a particular common scheme. The option 'Structure similarity' was used to remove dissimilar structures, and the option 'US-EPA New Chemical Categories' and 'Aquatic toxicity classification by ECOSAR' (Kaiser et al. 1999; Reuschenbach et al. 2008) were used to remove selected analogues. Other compounds that did not structurally match the targeted V-type NAs were manually removed to ensure that only appropriate structures were considered in the LD_50_ prediction.

### Application of Toxicity Estimation Software Tool (TEST)

The second applied software was the Toxicity Estimation Software Tool (TEST) (Gatnik and Worth 2010; Lapenna et al. 2010; Martin 2019), an open-source application developed by the US EPA. TEST (ver. 5.1.2 and ver. 4.2.1) comprises several models assessing acute toxicity thresholds (Diaza et al. 2015) by reading across structural analogues or multivariate regression. The models were built on hundreds of structural, constitutional, connectivity, shape, topological, molecular distance, fragments, and electrotopological property descriptors. The program demands only SMILES (Simplified Molecular Line Input System) (Toropov et al. 2005) or CAS numbers as inputs to evaluate chemical toxicity quickly. The software offers an estimated LD_50_ threshold based on each model prediction and a Consensus average of the component models. TEST assesses acute toxicity using four QSAR methodologies:

1. Consensus method—the predicted toxicity is estimated by taking an average of the predicted toxicities from each QSAR method's applicability domain; the Consensus result was reported as the most reliable estimate provided by the TEST software (Lunghini et al. 2019).
2. Hierarchical method—the toxicity for a particular query compound is estimated using the weighted average of the predictions from various models; the different models are achieved using Ward’s method to divide the training set into a series of similar structural clusters (Martin et al. 2008).
3. Nearest-neighbour method—the predicted toxicity is estimated by averaging the three chemicals in the training set with the closest similarity to the test compound (Chavan et al. 2015).
4. FDA method (only ver. 4.2.1)—the prediction for each test chemical is made using a new model that fits the chemicals most similar to the test compound. Each model is generated at runtime.

The advantages and disadvantages of the methods incorporated in TEST are described in a previous publication (Noga et al. 2023). Calculation options (end point: oral rat LD_50_, method: Consensus and FDA, relax fragment constraint: disabled, chemical transformation simulator: disabled).

### Application of ProTox-II

The ProTox-II (Drwal et al. 2014) web server is an open-access tool that can predict the toxicity of chemicals. The predictive capabilities solely rely on the two-dimensional structure of the input compounds (canonical SMILES) (Drwal et al. 2014; Banerjee et al. 2018a, b). Rigorous evaluation has been conducted using a diverse external validation set, demonstrating commendable performance. The sensitivity, specificity, and precision of the ProTox methods are reported as 76%, 95%, and 75%, respectively. The ProTox web server employs chemical similarity and identifying toxic fragments to predict toxicity accurately. Furthermore, it incorporates a unique feature of toxicity class prediction through similarity- and fragment-based methods, along with alerts indicating potential toxicity targets. One significant advantage of ProTox-II (Banerjee et al. 2018b) is its adaptability for future enhancements. A selected oral toxicity model based on a prediction method based on analysing two-dimensional similarity to compounds with known LD_50_ values and identifying fragments overrepresented in toxic compounds. The validation method is based on leave-one-out cross-validation. The three nearest neighbours from the training set are calculated for each compound using fingerprint similarity. Oral toxicity prediction results for the input compound are given as a predictive LD_50_ value (mg/kg).

### Validation of applied in silico methods

To achieve the appropriate validation of the applied in silico methods, we employed the only feasible strategy, which involved comparing results with known data of the same OPs. Specifically, we determined the prediction similarity index (%) considering the experimentally determined LD_50_ value for a given substance, which we treat as a reference value, in relation to the values estimated by the in silico method. For this purpose, OPs had to be selected that meet several criteria: (1) they have the same mechanism of action, (2) they undergo the same metabolism, (3) they do not directly belong to the same family of compounds that are subject to experimental studies, and (4) they have similar structure core. It should be noted that this is a challenging task in the context of the studied CWAs, as their number is strictly limited to a few (they are not broad groups of compounds like a given class of pesticides). Another problem is the lack of reliable and/or current experimental data. V-series chemical warfare agents are widely known, but experimental studies regarding their acute toxicity are (1) not widely available, (2) not currently determined by OECD guidelines, and (3) not a popular research topic. For this reason, there are only a few experimental data to which in silico study results can be compared. Given the imposed criteria for selecting substances for validation other than from the V-series but behaving analogously in terms of toxicology and with a similar structure, the only choices left were from the G-series, i.e. Tabun and Sarin. To address the proper classification of validated substances, we also chose a compound that can be classified as both V-series and G-series, i.e. VG. The validation results for the indicated substances using the applied methods are presented in Table 2.

**Table 2:** Summary of validation analysis

Compound		Method	Comment	Predicted (oral, rat) LD_50_ (mg/kg)	Experimental (oral, rat) LD_50_ (mg/kg)	Prediction similarity (%)
Tabun	CAS:	TEST Consensus	Same mechanism, same metabolism considerations	35.38	3.6 (Misik et al. 2015)	10
	77–81-6	TEST FDA		3589.37		0
	SMILES:	QSAR Toolbox		3.16		88
	N#CP(= O)(OCC)N(C)C	ProTox-II		4.00		90
Sarin	CAS:	TEST Consensus	Same mechanism, same metabolism considerations	ND	0.67 (Misik et al. 2015)	ND
	107–44-8	TEST FDA		527.36		0
	SMILES:	QSAR Toolbox		0.73		92
	O = P(F)(OC(C)C)C	ProTox-II		1.00		67
VG	CAS:	TEST Consensus	Same mechanism, same metabolism considerations	3.88	3.3 (ARSIM 1966)	85
	78–53-5	TEST FDA		0.41		12
	SMILES:	QSAR Toolbox		3.00		91
	O = P(OCC)(OCC)SCCN(CC)CC	ProTox-II		3.67		90

*ND* not determined

It should be noted that for V-series substances, validation should exclude using the TEST (consensus) method. However, due to the high prediction similarity index for the VX, we decided to apply this method for our purposes. The validation met the requirements in terms of conditions for determining LD_50_ (rat, oral) values, specifically oral administration in rats. It is important to note that other kinds of validation recommended by Kutsarova et al. (Kutsarova et al. 2021b) are not feasible due to the unique nature of chemical warfare agents, their limited quantity, and the criteria to fulfil validation conditions. The constraints arising from the specificity of the subject matter allow for validation to be conducted only in the manner we have undertaken.

## Results

The acute toxicity of the examined V-type compounds (*n* = 9), displayed as tLD_50_ values for oral administration to rats, was estimated using specialised software: QSAR Toolbox, TEST, and web server tool ProTox-II. Animal to human (rat-to-human) extrapolation was based on toxicity values conversed by the guidelines for converting doses between animals and humans, described by Nair and Jacob (by dividing the rat dose by 6.2 (Nair and Jacob 2016)). The estimated tLD_50_ values for the oral administration of V-agents and human-converted values are listed in Table 3.

**Table 3:** Rat and human oral LD_50_ values, calculated by TEST (ver. 5.1.2 and ver. 4.2.1), QSAR Toolbox (ver. 4.6) software, and ProTox-II tool

Number	Acronym	Rat oral LD_50_ (mg/kg bw.)				Human oral LD_50_ (mg/kg bw.)			
		QSAR Toolbox	TEST Consensus method	TEST FDA method	ProTox-II	QSAR Toolbox	TEST Consensus method	TEST FDA method	ProTox-II
1	VE	2.63	2.23	1.9	1.0	0.42	0.36	0.31	0.16
2	VG	3.67	3.88	0.41	3.0	0.59	0.63	0.07	0.48
3	VM	0.32	0.18	0.6	1.0	0.05	0.03	0.10	0.16
4	VR (RVX)	1.04	1.32	2.1	1.0	0.17	0.21	0.34	0.16
5	VS	3.65	4.12	1.38	1.0	0.59	0.66	0.22	0.16
6	V-sub x	6.88	6.05	5.99	3.0	1.11	0.98	0.97	0.48
7	VX	0.26	0.63	1.34	1.0	0.04	0.10	0.22	0.16
8	CVX	1.67	1.44	1.97	1.0	0.27	0.23	0.32	0.16
9	100A	12.9	9.28	22.8	1.0	2.08	1.50	3.68	0.16

Oral doses of tLD_50_ for rats, then converted to human doses, estimated by the Consensus method implemented in the TEST software, showed that the most dangerous of all examined V-series NA was the compound VM (**3**), whose value was 0.03 mg/kg bw. A slightly higher tLD_50_, reaching 0.1 mg/kg bw, was estimated for VX (**7**). Compounds VR (**4**) and CVX (**8**), structural VX isomers, reached tLD_50_ values of 0.21 mg/kg bw and 0.23 mg/kg bw, respectively. VE (**1**), the fifth most dangerous V-type NAs with acute toxicity of 0.36 mg/kg bw, differed only in an additional methyl group compared to the VM compound. VG (**2**), with a tLD_50_ value of 0.63 mg/kg, unlike the other tested V-agents, lacks a phosphonothioate functional group (any anion of the form R-O-PH(=O)-S- or similar forms having the negative charge on the oxygen atom) in its core, but a phosphorothioate. However, VS (**5**), a structure analogous to VE, differing in an additional methyl group at the carbon attached to the quaternary amine, was less toxic (0.66 mg/kg bw). The only structural difference that explains the variability in the half-life of the two compounds is due to electronic effects (electron-donating and/or electron-withdrawing groups) or steric effect (Yuan et al. 1990) associated with the additional methyl groups in the structure. The central functional group of the V-sub x (**6**) compound is based on phosphonothioate, which has been enriched with additional sulfur (aliphatic attachment), which certainly increases the tLD_50_ value of this NA to 0.98 mg/kg bw. However, the least toxic of the examined V-agents, with a tLD_50_ value of 1.50 mg/kg bw, is Substance 100A (**9**). This compound is the only V-series NA with a cycloalkane attached to a phosphorus–oxygen atom.

The FDA model deployed in TEST software computed the tLD_50_ values for each examined V-type NAs. The results for only two compounds (**1** and **8**) were approximately consistent compared to those estimated by the Consensus model. According to the FDA methodology, compound VG (**2**) had the lowest tLD_50_ value and 0.07 mg/kg bw. VM (**3**) reached a slightly higher value of 0.10 mg/kg bw, while in the Consensus model, it was the most dangerous compound (0.03 mg/kg bw). Interestingly, VX (**7**) and VS (**5**) appeared slightly less toxic NAs, reaching a tLD_50_ of 0.22 mg/kg bw. Values above 0.3 to 0.4 mg/kg bw were obtained for compounds VE (**1**), CVX (**8**) and VR (**4**). V-sub x (**6**) poses a risk about three times lower than the structures mentioned above (**1**, **4** and **8**), reaching the value: 0.97 mg/kg bw. Substance 100A (**9**), with a tLD_50_ value of 3.68 mg/kg bw. is the highest value, and thus the least toxic, of the V-series NAs examined.

The Consensus and FDA models demonstrated numerous inaccuracies in the tLD_50_ values for investigational V-series NAs. Therefore, we decided to additionally use the QSAR Toolbox to estimate the acute toxicity parameter. The tLD_50_ values, estimated using the QSAR Toolbox, correlated with the Consensus model of the TEST software for most of the analysed compounds. However, in the case of compounds VE (**1**) and V-sub x (**6**), for which the values from both TEST models are comparable, a greater correlation cannot be unequivocally stated. However, the tLD_50_ value estimated by the QSAR Toolbox for compound CVX (**8**) oscillates between the Consensus and FDA methodology results. The common feature of all the estimates is the highest tLD_50_ values, although still significant, are V-sub x (**6**) and Substance 100A (**10**). Considering this shift, the first five compounds with the lowest tLD_50_ values are analogous to the Consensus method.

Additionally, the online tool ProTox-II was used to predict the oral toxicity of rodents, and then rat doses were converted to human doses. Compounds VG (**2**) and V-sub x (**6**) reached tLD_50_ values of 0.48 mg/kg bw, while the rest of the examined V-agents reached 0.16 mg/kg bw. The acute toxicity model of the ProTox-II platform had lower predictive accuracy than the other models described earlier; correlations can only be found forcibly with the VG (**2**) compound. However, for the remaining eight examined NAs, the obtained values differ entirely from the estimations obtained with previous software.

## Discussion

The results of the FDA and Consensus methods differ significantly, so the question is which model is more reliable? The FDA method is reinforced by new models generated based on the closest analogues of the substance tested. However, for some reason, the latest version of the TEST software (5.1.2) does not include the FDA model implemented, only available in the earlier version (4.2.1). In contrast, the Consensus model utilises all QSAR methods included in the TEST software for toxicity assessment. Moreover, the Consensus model was reported as the most reliable estimation method provided by the TEST software (Melnikov et al. 2016). Furthermore, the values of acute toxicity parameters obtained using the QSAR Toolbox are overwhelmingly correlated with the Consensus method; no compounds of the V series had a value closer to the FDA method. The only compounds whose tLD_50_ values in both TEST models are used in some way similar are VE (**1**) and V-sub x (**2**). Considering the arguments, the Consensus method and QSAR Toolbox results are more robust. Unfortunately, the LD_50_ parameter is given mainly for the VX compound and its structural isomers RVX and CVX by literature sources.

The lack of correlation with previously used software and models and the similarity between the values of the assessed V-series NAs most likely result from the operation of ProTox-II. The web server provides rodent oral toxicity prediction based on similarity analysis of compounds with known tLD_50_ and identification of toxic fragments. In the absence of values implemented in databases, as is the case with NAs, most of the results obtained are the same (1 mg/kg bw or 3 mg/kg bw) because they are likely based on the similarity of the same structures provided by the database.

The acute toxicity of the VM (**3**) estimated in our work for oral administration to rats, obtained using the Consensus model, is 0.18 mg/kg bw. It correlates to a large extent with the values published in the work of Bajgar, where the LD_50_ is 0.212 mg/kg bw (Bajgar 2004). Based on literature data, the LD_50_ values for VX (**7**) are 0.09 mg/kg bw (Bajgar 1985, 1991; Marrs et al. 1996) and 0.12 mg/kg bw (Misik et al. 2015) when administered orally to rats. These data differ slightly from our estimated value of 0.26 mg/kg bw using the QSAR Toolbox. However, the extrapolated acute toxicity values for VX from animals to humans were estimated at 0.10 mg/kg bw (Consensus TEST) and 0.04 mg/kg bw (QSAR Toolbox). The obtained values were fully correlated with the data in the literature, where the tLD_50_ for oral administration to humans was between 0.04 and 0.14 mg/kg bw (Moyer et al. 2014), and toxicity assessed for humans was 0.07 mg/kg bw (Bajgar 1985, 1991; Marrs et al. 1996). The acute toxicity of the VG (**2**), orally administered to rats, at the value of 3.67 mg/kg bw, was somewhat contradicted by data from (Moyer and Salem 2014), where the LD_50_ was 5.4 mg/kg bw. However, another source where the toxicity value was 3.3 mg/kg bw confirms our estimation (ARSIM 1966). For the compound VR (**4**), two distinct acute toxicity values were found in the literature review when administered orally to rats: 1.4 mg/kg bw (Misik et al. 2015) and 0.55 mg/kg bw (Zhukov et al. 2007). The first value almost perfectly correlates with the result obtained by the Consensus method (1.32 mg/kg bw), while the second value is less than twice the estimated value. Other published experimental data related to the acute toxicity of V-type NAs are included in Supplementary Materials 1 (SM1).

Extrapolation of doses between species is particularly worth looking at. The allometric approach accounts for differences in body surface area related to animal weight while extrapolating doses between species. In our work, rat doses were converted to human equivalent doses by dividing the rat dose by 6.2 (Nair and Jacob 2016). The allometric scaling of different species to convert doses from animal to human studies is considered one of the most controversial areas of pharmacology and toxicology. Science changes in phases, experiencing a series of anomalies that lead to crisis and revolution. The result is a novel, immature scientific paradigm that becomes the new norm (Hartung 2021). One of these crises is the guide to converting doses between species, which is not necessarily right. Make it clear that in toxicology, humans are not 70 kg mice (Leist et al. 2008). This is evidenced by a study based on a broad system approach that confirms the low predictability of animal responses to inflammation (Seok et al. 2013). The low levels of predictability when directly comparing data between species raises severe doubts about the usefulness of animal data as essential tools for predicting human safety. Most likely, that is the reason for variations in predictions, or maybe is it alternative evidence of the validity of Hartung's concept? (Hartung 2009, 2021). Notwithstanding the foregoing, these studies were necessary as an initial screening test prior to performing acute toxicity animal studies with V-series NAs.

## Conclusions

OP-CWAs pose a severe threat to life. In particular, V-series NAs pose a constant danger, as evidenced by examples of using them so far, such as: during the Angola Civil War (Hawk et al. 2014), the Halabja chemical attack (CIA 2007; Hiltermann 2007), action by Aum Shinrikyo (Nakagawa and Tu 2018) and assassination of Kim Jong-nam (Tu 2020). Due to their extreme toxicity, the threat posed by V-agents needs to be urgently assessed to be able to deal with future terrorist attacks or the use of chemical weapons on the battlefield. Furthermore, the use of lethal chemicals defined in the Chemical Weapons Convention (CWC) should be constantly monitored. Therefore, some light has been shed on the acute toxicity of V-type NAs by estimating the tLD_50_. Estimation was performed using in silico software: Toxicity Estimation Software Tool (TEST), QSAR Toolbox, and ProTox-II. According to our assessments, the most lethal V-agents were VX (**7**), VM (**3**) and structural VX analogues: VR (**4**) and CVX (**8**), tLD_50_ values (administered orally) were 0.04 mg/kg bw, 0.05 mg /kg bw, 0.17 mg/kg bw and 0.27 mg/kg bw, respectively. The least dangerous compounds were V-sub x (**6**) and Substance 100A (**9**), whose acute toxicity values reached 0.98 mg/kg bw and 1.50 mg/kg bw. Further in silico studies of various properties (chemical, physical, or toxicological) are needed to deal with the inevitable use of NAs in terrorist attacks. Our toxicology findings provide the first comprehensive information on the acute toxicity (LD_50_, rat, oral) of many V agents (*n* = 9). The TEST and QSAR Toolbox software can successfully estimate the tLD_50_ of V-series OPs prior to experimental laboratory tests.

### Supplementary Information

Below is the link to the electronic supplementary material.

Supplementary file1 (PDF 184 KB)

## Data Availability

All data generated or analysed during this study are included in this published article.

## Abbreviations

bw: Body weight
CAS: Chemical Abstracts Service
CWA: Chemical warfare agent
LD_50_: Median lethal dose
tLD_50_: Theoretical-median lethal dose
NA: Nerve agent
OP: Organophosphate
OP-CWAs: Organophosphorus chemical warfare agents
OECD: Organization for Economic Cooperation and Development
QSAR: Quantitative structure–activity relationship
SMILES: Simplified Molecular Line Input System
TEST: Toxicity Estimation Software Tool
TTC: Threshold of toxicological concern
