# Supplementary material for: The prediction of acute toxicity (LD50) for organophosphorus-based chemical warfare agents (V-series) using toxicology in silico methods
Source: Arch Toxicol. 2023 Dec 5;98(1):267–75. doi: 10.1007/s00204-023-03632-y (PMC10761519; doi:10.1007/s00204-023-03632-y)
Supplement: Supplementary file 1 — Supplementary file1 (PDF 184 KB) [file 204_2023_3632_MOESM1_ESM.pdf]

Supplementary Materials 1 (SM1)

1. Other published experimental data related to acute toxicity of V-type nerve agents.

Percutaneous route:

- Guinea-pigs (*n* = 8) died after being poisoned with the VX (0.74 mg/kg) (~2.5 × LD_{50}). All guinea-pigs treated with Protexia (*n* = 8) survived. Protexia® is a pegylated form of recombinant human butyrylcholinesterase (Mumford and Troyer 2011).

- Application of VX (0.592, 0.296, 0.148 mg/kg) to guinea-pigs (*n* = 4 per dose). Three out of four animals died within 24 hours following the highest dose. One animal was still alive at 24h, although it was substantially incapacitated. The lower two doses of VX produced extended periods of bradycardia without observable signs of poisoning (Mumford et al. 2008).

- Percutaneous, LD_{50} – rabbit (0.014 mg/kg), cat (0.01 mg/kg), dog (0.0157 mg/kg), mouse (0.016 mg/kg) and human (0.1-0.01 mg/kg) (Rembovskiy et al. 2020). Important, not indicated in the original study.

Subcutaneously route:

- Conscious or anaesthetised beagle dogs were subcutaneously injected with VX (1.5, 3.0 or 6.0 mg/kg b.w.). Blood cholinesterase activity decreased to 60%, 20% and 18% respectively of initial values. Only in the 6.0 mg/kg-treated group heart rate, arterial and left intraventricular pressures, and contractility index significantly decrease (Robineau and Guittin 1987). Therefore, it is assumed that subcutaneous LD_{50} in beagle dogs is 6 mg/kg.

Dermal route:

- Domestic swine model, 6h LD_{50} for VR topically applied on the ear was 100 µg/kg (Mikler et al. 2011).

- Male York-Landrace cross pigs were exposed to 2× (124 ug/kg) or 5× LD50 (310 ug/kg) of neat VX applied topically onto the ventral surface of the ear. Animals treated with either 2× or 5× LD50 of topically applied VX exhibited typical signs of OP nerve agent poisoning and died within ~70–100 min (Sawyer et al. 2011).

- Dermal, airborne LCt_{50} – dog clipped (4.6 mg-min/m^3), rabbit clipped (8.3 mg-min/m^3) and rabbit clipped, clothed (539 mg-min/m^3) (Munro 1994).

- Dermal, liquid LD50 – monkey shaved (0.065 mg/kg), pig clipped (0.40 mg/kg), dog depilated (0.054 mg/kg), cat depilated (0.012 mg/kg), rabbit depilated (0.025 mg/kg), rat depilated (0.10 mg/kg), and mouse depilated (0.046 mg/kg) (Munro 1994).

Inhalation route:

- VX-exposed guinea pigs, 27.03 mg/m3 of VX using a microinstillation inhalation exposure technique for 4 min. Exposure to this dose of VX resulted in a 24-hour survival rate of 52% (Nambiar et al. 2007).

- Inhalation, LCt_{50} – monkey (50 mg-min/m^3), dog (15 mg-min/m^3), rat (17 mg-min/m^3) and mouse (40 mg-min/m^3) (Munro 1994).

- Inhalation, LCt_{50} – mouse (0.011 mg-min/ m^3) (Rembovskiy et al. 2020).

- LCt_{50} and LC_{50} in male and female adult SD rats exposed to whole-body VX vapour for 10, 60, and 240 min in a dynamic exposure chamber. LC_{50} (mg/m^3), VX, whole body (M/F) = (0.16/0.16), (0.65/0.74), (4.85/5.44), respectively for 240, 60 and 10 min exposure. LCt_{50} (mg-min/m^3) VX, whole body (M/F) = (39.6/39.4), (39.2/44.4), (48.5/54.4), respectively for 240, 60 and 10 min exposure (Benton et al. 2006; Young and Watson 2020).

Intravenous route:

- LD_{50} – monkey (0.0084 mg/kg), goat (0.005 mg/kg), dog (0.0063 mg/kg), cat (0.0025 mg/kg), rabbit (0.0084 mg/kg), rat (0.0079 mg/kg) and mouse (0.0141 mg/kg) (Munro 1994).

2. Supplementary references:

Benton BJ, McGuire JM, Sommerville DR, et al (2006) Effects of Whole-Body VX Vapor Exposure on Lethality in Rats. Inhalation Toxicology 18:1091–1099. https://doi.org/10.1080/08958370600945598

Mikler J, Tenn C, Worek F, et al (2011) Immobilization of Russian VX skin depots by localized cooling: Implications for decontamination and medical countermeasures. Toxicology Letters 206:47–53. https://doi.org/10.1016/j.toxlet.2011.05.1047

Mumford H, Price ME, Wetherell JR (2008) A novel approach to assessing percutaneous VX poisoning in the conscious guinea-pig. Journal of Applied Toxicology 28:694–702. https://doi.org/10.1002/jat.1324

Mumford H, Troyer JK (2011) Post-exposure therapy with recombinant human BuChE following percutaneous VX challenge in guinea-pigs. Toxicology Letters 206:29–34. https://doi.org/10.1016/j.toxlet.2011.05.1016

Munro N (1994) Toxicity of the Organophosphate Chemical Warfare Agents GA, GB, and VX: Implications for Public Protection. Environmental Health Perspectives 102:18–37. https://doi.org/10.1289/ehp.9410218

Nambiar MP, Gordon RK, Rezk PE, et al (2007) Medical countermeasure against respiratory toxicity and acute lung injury following inhalation exposure to chemical warfare nerve agent VX. Toxicology and Applied Pharmacology 219:142–150. https://doi.org/10.1016/j.taap.2006.11.002

Rembovskiy V, Savelieva E, Radilov A, et al (2020) Chapter 9 - Russian VX. In: Gupta RC (ed) Handbook of Toxicology of Chemical Warfare Agents (Third Edition). Academic Press, Boston, pp 127–141

Robineau P, Guittin P (1987) Effects of an organophosphorous compound on cardiac rhythm and haemodynamics in anaesthetized and conscious beagle dogs. Toxicology Letters 37:95–102. https://doi.org/10.1016/0378-4274(87)90173-1

Sawyer TW, Mikler J, Worek F, et al (2011) The therapeutic use of localized cooling in the treatment of VX poisoning. Toxicology Letters 204:52–56. https://doi.org/10.1016/j.toxlet.2011.04.008

Young RA, Watson A (2020) Chapter 8 - Organophosphate nerve agents. In: Gupta RC (ed) Handbook of Toxicology of Chemical Warfare Agents (Third Edition). Academic Press, Boston, pp 97–126
